# Huntington’s Disease—An Outlook on the Interplay of the HTT Protein, Microtubules and Actin Cytoskeletal Components

**DOI:** 10.3390/cells9061514

**Published:** 2020-06-22

**Authors:** Aleksandra S. Taran, Lilia D. Shuvalova, Maria A. Lagarkova, Irina B. Alieva

**Affiliations:** 1 Faculty of Bioengineering and Bioinformatics, Lomonosov Moscow State University, 1–73, Leninsky Gory, 119992 Moscow, Russia; eva8326@yandex.ru (A.S.T.); shuvalova_l@inbox.ru (L.D.S.); 2 Federal Research and Clinical Center of Physical-Chemical Medicine of Federal Medical Biological Agency, 1a Malaya Pirogovskaya St., 119435 Moscow, Russia; 3 Center for Precision Genome Editing and Genetic Technologies for Biomedicine, Federal Research and Clinical Center of Physical-Chemical Medicine of Federal Medical Biological Agency, 1a Malaya Pirogovskaya St., 119435 Moscow, Russia; 4 A.N. Belozersky Institute of Physico-Chemical Biology, Lomonosov Moscow State University, 1–40, Leninsky Gory, 119992 Moscow, Russia

**Keywords:** neurodegenerative diseases, proteinopathies, Huntington’s disease, cytoskeleton, microtubules

## Abstract

Huntington’s disease is a severe and currently incurable neurodegenerative disease. An autosomal dominant mutation in the Huntingtin gene (*HTT*) causes an increase in the polyglutamine fragment length at the protein N-terminus. The consequence of the mutation is the death of neurons, mostly striatal neurons, leading to the occurrence of a complex of motor, cognitive and emotional-volitional personality sphere disorders in carriers. Despite intensive studies, the functions of both mutant and wild-type huntingtin remain poorly understood. Surprisingly, there is the selective effect of the mutant form of HTT even on nervous tissue, whereas the protein is expressed ubiquitously. Huntingtin plays a role in cell physiology and affects cell transport, endocytosis, protein degradation and other cellular and molecular processes. Our experimental data mining let us conclude that a significant part of the Huntingtin-involved cellular processes is mediated by microtubules and other cytoskeletal cell structures. The review attempts to look at unresolved issues in the study of the huntingtin and its mutant form, including their functions affecting microtubules and other components of the cell cytoskeleton.

## 1. The Cytoskeleton Plays an Important Role in the Neuronal Morphology, Plasticity, and Neurodegenerative Processes

Neurons are the main units of reception, procession, and transmission of information in the nervous system. They establish connections using long specialized processes—unbranched axons, whose length can exceed a meter, and shorter branched dendrites. The cytoskeleton is the main intracellular player that determines the shape of the cell. In neurons, the cytoskeleton consists of three filament systems—microtubules (MTs), microfilaments, and neurofilaments. Unlike neurofilaments, tubulin-polymerized MTs and actin-polymerized microfilaments are highly dynamic structures, which determines their important role in the development of neurons and their plasticity, and neurodegenerative processes [1]. Besides, MTs and microfilaments are polar structures; they provide pathways for motor protein-mediated directional transport, which is of particular importance for neurons with long axons. Various associated proteins modulate the dynamicity of the MT and actin microfilament system, shifting the balance of the polymerization reaction, which leads either to the formation of a polymer or to the formation of monomers of the corresponding proteins. In addition, several factors act as cross-linkers of the two filament systems, providing a mechanism for their property changes, interrelationship within a wide range of functions. Morphological changes during the development and functioning of the nervous system are the result of a complex interplay of the MT system and actin filaments in the neuron [2], and in this sense, the neuron is no different from other types of cells [3,4,5].

## 2. Microtubules (MTs) and Their Properties Necessary for the Neuron’s Development and Functioning

MTs are hollow non-branching tubes of various lengths with a diameter of about 25 nm. Thirteen laterally linked protofilaments, consisting of α, β-tubulin dimers [6] joined with each other by the “head to tail” principle [7] are forming the MT 5 nm thick wall. As a consequence of “head to tail” connections MT is polar structure—due to the linear polymerization of α- and β-tubulin heterodimers, α-tubulins of each dimer (minus end) are located at one end of the protofilament, and β-tubulins (plus-end) are disposed at the other end.

The addition of tubulin subunits occurs predominantly at the plus-end of MT [8]. It was shown that MTs are dynamic structures, their assembly phases alternate with disassembly phases, and this behavior termed “dynamic instability” [9,10,11]. MT dynamic instability has the following parameters: MT polymerization (growth) and depolymerization (shrinkage, shortening) rates, catastrophe (transition from polymerization to depolymerization) and rescue (transition from depolymerization to polymerization) frequencies, as well as pauses—states when MTs are not polymerized or depolymerized [12]. All parameters are finely regulated in a cell; the polymerization and depolymerization rates of individual MT can vary significantly both in cell and in different types of cells [13,14,15].

The polarity and dynamic instability are properties of MTs critical for cell life. MTs are key participants in such cellular processes as membrane vesicle and organelle transport, DNA segregation by the mitotic spindle during mitosis, cell migration, and cellular polarity maintenance. Neurons are complex polarized cells. The specificity of their morphology allows them to establish intercellular connections and conduct chemical and electrical signals. MTs are an important structural component responsible for the morphology of neurons necessary for signal reception and transmission. MTs are necessary for the development of the nervous system and the implementation of numerous neuron-specific processes, especially for intracellular chemical transport over long distances [16,17,18].

**Figure 1:**
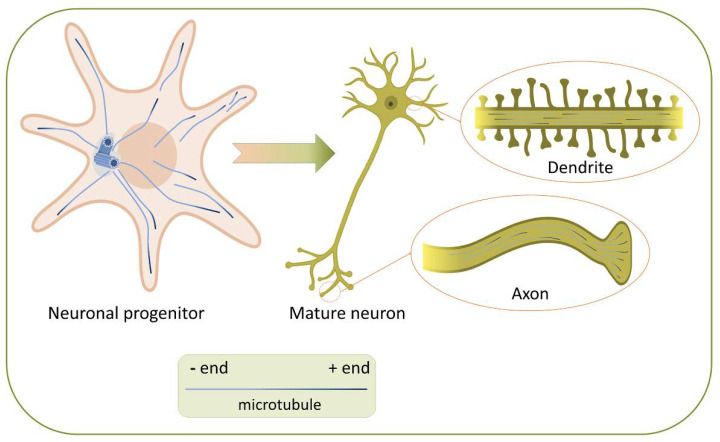
Microtubule organization in neuronal progenitor and mature neuron. In the early stages of neuron polarization, MTs originate from the centrosome, where their minus-ends are located during nucleation and elongation before detachment from the centrosome (or oriented after detachment). Hence, the structure of MTs in neuronal progenitor is radial. When neurites mature into axons and dendrites, the structure of the MT network changes. In dendrites, MTs became antiparallel: unique feature of the dendritic MT cytoskeleton is the appearance of MTs with their minus-ends oriented towards the dendritic tips. Simultaneously, in the axons, all the minus-ends are directed towards the body of the neuron.

The cytoskeleton and the MT system play an active role during various phases of neuron polarization. In young neurons - spherical unpolarized cells, the MT system is radially organized. MTs are growing mainly from the centrosome: their minus-ends are inside, and the plus-ends oriented to the cell periphery [19]. During neuronal differentiation, the symmetry breaks, membrane protrusions begin to form, which lengthen, forming thin outgrowths called neurites [20]. These outgrowths mature into axons and dendrites when the neuron is polarized (Figure 1). Recent decades have convincingly demonstrated that MTs are necessary both for the neurites formation [21] the axons outgrowth [22,23], dendrites [24] and synapses [25,26]. As previously shown, both dendrites and axons contain a fraction of dynamic MTs [27,28,29,30]. Later, it became possible to measure the dynamics of MT polymerization. MT polymerization rates are similar in axons and dendrites of primary hippocampal neurons and Purkinje neurons in vitro [31], in dendrites of mature cultured primary hippocampal neurons [25], as well as in dendrites of cortical neurons in the brain of adult mice [24,32].

Due to their ability to change dynamic properties and stabilize, MTs direct specific membranetraffic and affect actin dynamics by determining axon formation, maintain axon identity, and regulate the dynamics of dendritic spines [1,33,34]. 

The interaction between actin and MTs by cross-linker proteins is very important for establishing the complex polarized morphology of neurons. During the formation of axons, actin filaments are indirectly stabilized by MTs [35], especially in the initial segment of axon, which is the unique neural compartment, playing a decisive role in generating the action potential and polarity of neurons [36]. Axon forms after neurite expansion due to the local stabilization of MTs in pre-axonal neurites [22]. The process of elongation of a newly formed axon directly depends on stable MTs, which form “rails” for the transport of proteins and organelles necessary for the formation of new axonal segments [23]. The formation of dendrites starts with the dramatic reorganization of MTs in precursor neurites—even before the beginning of dendritogenesis, neurites undergo several cycles of alternating phases of growth and shrinkage, which establish the orientation of approximately 80% of MT plus-ends to the cell periphery [24]. A striking feature of the dendritic MT cytoskeleton organization is the appearance of MTs with their minus-ends oriented towards the dendritic tips [27,28]. Therefore, dendrites acquire unique antiparallel bundles of MTs (plus and minus ends distal) whereas MTs are parallel in axon (plus ends distal). Differences in the MT orientations between axon and dendrites probably contribute to the correct “targeting” of a specific cargo providing the possibility of transport by certain motor proteins.

Differences in the MT orientations between axon and dendrites likely contribute to the correct “targeting” of a specific cargo by enabling transport by specific motor proteins

The formation of synapses—connections between an axon of one neuron and a dendrite of another, is the final and ongoing step in neuronal maturation. Excitatory synapses are formed on dendritic spines, which are actin-rich protrusions on the dendrite. Dynamic MTs contribute to the formation of dendritic spines; they can penetrate them temporarily, polymerizing in proximal regions [25,26]. MTs and their dynamic behavior affect not only the formation and functioning of neurons and a healthy brain but also the processes of brain aging and the development of neurodegenerative diseases [1,37]. Many neurological diseases arise due to defects in MTs and other components of the cytoskeleton and/or their regulation [1,35].

## 3. The Role of Endogenous MT Modulators and Tubulin Post-Translational Modifications in the Development of the Nervous System Diseases

Since the development of the brain largely depends on the normal functioning of the cytoskeleton, defects of the latter can lead to fatal consequences for the proliferation, migration, and communication of neurons. Mutations affecting MTs are associated with various disorders in the nervous system development, including lissencephaly, microcephaly, polymicrogyria, autism spectrum disorders, and intellectual disorders [38,39,40]. Mutations in MT-associated proteins can alter their functions in neurons [41]. The published data suggest that HTT is capable of regulating the switching of vesicle transport from MTs to actin filaments [42,43]. There is a possibility that mutant HTT (mHTT) cannot carry out this switching normally, which is one of the plausible reasons for the breach in vesicular transport. The main endogenous factors modulating the dynamic properties of MTs are MT-associated proteins: (1) proteins interacting with MT ends, and (2) proteins interacting with the lateral surface of MTs. Post-translational modifications of tubulin make a significant contribution to the variability in MT dynamics. 

Proteins interacting with MT ends—MT plus-end tracking proteins—structurally and functionally diverse regulatory factors, located at the growing MT plus-ends which can form complex macromolecular assemblies. MT plus-end proteins regulate the dynamics of MTs, as well as facilitate their interaction with other proteins, organelles, and actin structures [44]. Studies indicate that many families of MT plus- end tracking proteins are crucial for various processes of the nervous system development, especially for the formation of neurites and axons. The main families of MT plus- end tracking proteins involved in the development of neurons are the proteins of: (1) EB family, which regulate MT during the formation of axons and dendrites [45,46]; (2) APCs, that playing an important role in establishing neuronal polarity and regulating axon migration and navigation by stabilizing MTs [47,48,49]; (3) CLIPs, regulating axon formation and growth, as well as dendritic branches growth [35]. The role of minus-end tracking proteins is not completely understood. While the plus-ends of MTs are highly dynamic, the minus-ends are stable and often capped in neurons. Several recent studies have shown that the minus-end tracking protein CAMSAP2 controls the organization of antiparallel MTs network in dendrites [50], and the slow processive growth of free minus-ends [51]. The possible differences in MT dynamics in wild-type and *HTT* mutant cells have not been studied to date, and it is very interesting to investigate the composition of their regulatory MT ends protein spectrum.

The other group of MT-associated proteins—MT lattice-binding proteins (MAPs—MAP1A, MAP1B, MAP2, and tau) interact with the lateral surface of MTs, stabilize MTs, and regulate their dynamics during the development of neurons. MAP1B is predominantly expressed in the early stages of maturation, while tau and MAP2 are expressed in both immature and mature neurons [52]. MAP2A and MAP2B are predominantly localized in the cell bodies and dendrites of mature neurons [53] while tau propagates mainly in axons [54] and dendrites [55]. Unlike other MT-associated proteins, the role of tau protein in the development of pathology in the brain of some patients with Huntington’s disease (HD) is clear [56], while the role of mHTT in the disease progress is not known [57,58,59].

Post-translational modifications of tubulin can regulate the dynamics and mechanical properties of MTs and their interaction with various proteins, such as molecular motors. Stable MTs enriched in acetylated tubulin are located in both dendrites and axons [60]. However, in cultured mature neurons, the level of modified tubulin is higher in axons than in dendrites [61,62]. The MTs enriched in de-tyrosinated tubulin are located in proximal axon segments [30], whereas MTs with tyrosinated tubulin in cell bodies and dendrites [63]. Strikingly, excessive polyglutamylation of MTs leads to neurodegeneration in mice and humans. The possible reason is the disturbances in axon microtubule transport, causing cell death [64,65,66]. Our preliminary observations indicate that MTs in wild-type and *HTT* mutant cells consist of tubulin with different post-translational modifications specific for each *HTT* variant. However, these findings require further investigation. 

Thus, MT organization and their dynamic properties are important for the normal neurons’ maturation and functioning.

## 4. Huntington’s Disease—A History of Studies and Unanswered Questions

**Figure 2:**
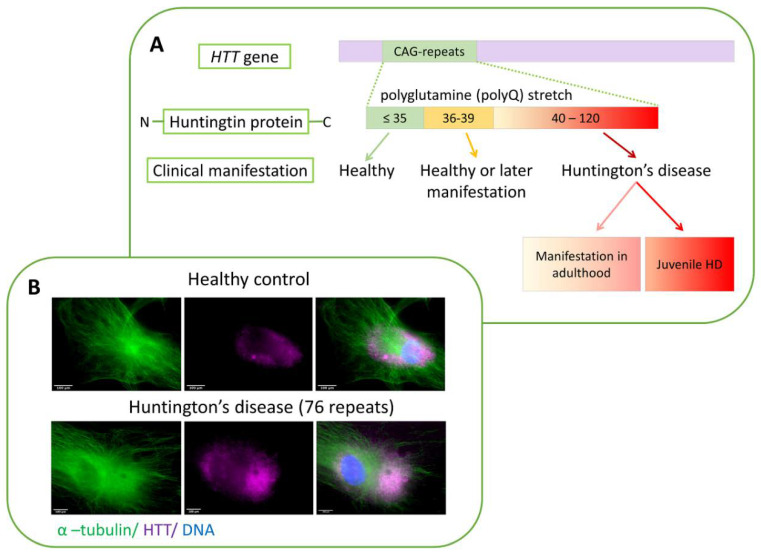
(**A**) The correlation of the number of CAG repeats in the *HTT* gene with the clinical manifestation of HD. The number of repeats ≤35 is normal; from 36 to 39 may not phenotypically manifest (or lead to the development of HD at a later age); from 40 upwards in 100% of cases causes HD. Hence, the age of disease onset and the severity of clinical symptoms depend on the number of CAG repeats according to the inverse correlation principle. (**B**) Immunocytochemical detection of HTT in the skin fibroblasts of healthy donors and patients with HD. Localization of HTT in the cells of a healthy donor (top panel) and a patient with HD (bottom panel).

Neurodegenerative diseases comprise an extensive list of pathologies associated with the gradual degradation and death of certain types of neurons. One of the groups of such pathologies, polyglutamine diseases, includes Huntington’s disease (HD). HD is a hereditary progressive autosomal dominant neurodegenerative disease [67,68], which is characterized by cognitive, motor, and psychiatric disorders. A mutation in the first exon of the gene coding for a protein called huntingtin (HTT) [69] causes the disease. Cytogenetic location of *HTT*: 4p16.3, which is the short (p) arm of chromosome 4 at position 16.3. Homologs of this gene exist in many organisms distant from mammals in evolution, which indicates the special importance of HTT for cell physiology [70]. As a result of the mutation, a multiplication of the CAG codon encoding the amino acid glutamine occurs [71]. A mutation in the *HTT* gene belongs to dynamic mutations: the first exon contains a sequence of tandem CAG repeats, the number of which can vary over a fairly wide range. HD starts to develop when the number of CAG triplets exceeds 35 (Figure 2A) [72].

It is plausible that the reason for this type of mutation is the replication errors happening during meiosis, which are more likely on repeated DNA sequences [73]. The incidence rate of the disease in the world is approximately 5 per 100,000 people [74]. Among the clinical manifestation chorea, tremors, decreased attention, communication disorders, dementia, depression, aggression, and other symptoms were described [75]. The debut of the disease most often occurs when patients are 30–50 years old, although cognitive decline and behavioral disorders can manifest much earlier. The age of development and the severity of physical and mental symptoms are directly related to the number of CAG repeats. However, family members and even individuals with the same number of repeats (including identical twins) can have significant variability both in the time of the onset of the first signs of the disease and in the intensity of its symptomatic manifestations [72,76,77]. These facts indicate the existence of currently unknown factors affecting the development and severity of HD [78]. At the moment, there is no effective way to treat HD (only symptomatic treatment is available in clinical practice). Therefore, 10–15 years after the first signs of the disease appear, patients die. 

The *HTT* gene expresses in all tissues and organs in humans and mice. Of importance, the level of *HTT* expression is the highest in neural tissue [79]. *HTT* knockout mice are embryonic lethal because it causes significant gastrulation defects [77,80,81]. The structure and domain organization of HTT is still not fully described and remains the subject of research. The N-terminal region of the protein has been the most studied since it contains the polyglutamine fragment.

The number of HTT partner proteins is quite large, and according to various researchers, there can be from 100 to 350 [82,83,84]. Such a large number of molecular interactions, the nature of the partner proteins, as well as the relatively large size and stability of HTT suggest that it acts as a kind of scaffold. The presence of HEAT (Huntington, Elongation Factor 3, PR65/A, TOR) domains makes it even more plausible. Thus, a possible function of HTT might be coordinating cellular processes as a central scaffolding component of many protein complexes [43].

Macroscopically, HD neuropathy manifests itself in bilateral atrophy of certain regions of the brain, in particular the striatum [85,86]. First of all, medium spiny GABA-ergic (gamma-aminobutyric acid) neurons are affected [87,88]. With the progression of the disease, at its later stages, death of the substantia nigra [89], cerebral cortex [87,90,91], globus pallidus neurons [92], and other parts of the brain occur. Along with neuronal changes, researchers note a reaction from neuroglia, in particular reactive gliosis (proliferation and activation of glial cells), which the authors consider to be part of the inflammatory response [93]. 

In addition to the death of neurons in various parts of the brain, patients also show degenerative changes in other tissues: decreased muscle mass, cardiac abnormalities, and pathologies in the gastrointestinal tract [94]. Hence, it is obvious that with the development of Huntington’s disease: (1) nervous tissue is most critically affected; (2) disorders, although less pronounced, affect other types of tissue. The selective effect of the mutant form of HTT on neural tissue is a separate scientific problem [79]. At the moment, this problem has no solution, because the cellular functions of the wild-type of HTT remain poorly understood. Nevertheless, the results of HD studies allow us to outline the range of intracellular processes in which HTT is involved: firstly, vesicular [95,96] and organelle transport [97], endocytosis [98]; secondly, the process of autophagosome formation, degradation of proteins and whole organelles [99,100,101]; thirdly, mitosis [102,103]; and lastly, primary cilia formation process [43,104]. It looks like most of the cellular processes in which HTT is involved are associated with MTs and actin and/or mediated by them [42].

## 5. Intracellular Localization of Huntingtin and Its Association with the Cell Cytoskeleton

Experimental studies have shown the diffuse distribution of the wild-type HTT in the cell cytoplasm, concentrating around the nucleus, in glia cells, and neurons of huntingtin transgenic mice [105,106,107]. mHTT is located mainly in the nuclei of neurons and glial cells [108]. Moreover, the N-terminal fragments of mHTT after proteolysis co-locate with the nucleus [109]. Furthermore, in the culture of primary fibroblasts obtained from the skin of both healthy donors and patients with HD, HTT diffusely locates in the cytoplasm around the cell nucleus (Figure 2B).

Data on HTT distribution motivated further studies to search for the direct interaction of HTT with the components of the cytoskeleton. However, at present, this issue remains debatable. It is plausible that the perinuclear localization of HTT may reflect interaction with β- and γ-tubulin [110]. Studies show that in normal conditions, HTT co-locates with MTs. Protein concentration is higher in the central region of the cell. That is exactly the place where MTs converge in the cell. Moreover, after exposure to nocodazole (MT-depolymerization agent), the distribution of HTT has become diffuse.

There are experimental evidences of a possible association of HTT with actin cell structures and focal contacts. Thus, mHTT co-localizes with actin in the lamellipodia of mouse cells [111]. In further experiments, exogenously expressed HTT fragment (1–558 aa) fused to GFP, F-actin, and BAIAP2 co-located with the filopodia of cultured NIH3T3 mouse fibroblasts [83].

Previous proteomic experiments have shown that HTT interacts with α-actinins 1, 2, and 4 [83,112]. However, immunofluorescence studies have not confirmed those findings. The functional interaction of HTTs with α-actinin isoforms has confirmed by the integrated approach—immunoprecipitation method using exogenously expressed proteins in combination with immunofluorescence and PLA analysis [113]. The authors suggest that HTT regulates the localization of α-actinin-1 and combines growth factor signaling with actin polymerization and the formation of actin bundles in newly formed adhesion sites. Therefore, the results of recent studies indicate the relationship of HTT with individual components of the cytoskeleton of the cell, including MTs and actin filaments.

## 6. HTT Involvement in Cell Life Processes: Normal Protein Functions and Toxicity of the Mutant Form of HTT

There are several hypotheses on the effect of an extended polyglutamine fragment on cell physiology. In particular, there is a model of mHTT conformation changes in the presence of a long polyglutamine fragment. The polyglutamine repeat (17–34 repeats) between the N-end domain (17aa) and the polyproline domain of wild type HTT acts as a flexible hinge. That allows the amino-terminal domain to interact with the downstream polyproline one, enhancing the interaction of HTT with the cell membrane. More repeats in a mHTT lead to a decrease in the flexibility of the hinge, disrupting the interaction of domains [114].

The mHTT is toxic due to aggregation and accumulation of an excess protein in cells. The lysosomal system removes toxic forms from cytoplasm via macrophages and HTT acetylation. In the nucleus, wild type and mHTT undergo proteolysis. In the cytoplasm, the dynamic interaction of HTT with the endoplasmic reticulum leads to the formation of autophagosomes. The association of HTT with late endosomes, autophagic vesicles, Rab5, indirectly indicates that one of the normal functions of HTT is autophagy. Interestingly, this includes the autophagy of mHTT by wild type one [99,100,101]. During autophagy, HTT promotes p62-mediated recognition of cargo, as it increases the affinity between p62 and ubiquitinated protein fragments [115,116].

Autophagosome formation is abnormally high in the cells of HD patients. Moreover, mHTT has a dual effect on the normal course of this process. Firstly, mHTT enhances autophagy via the inactivation of mTOR kinase [117,118]; secondly, HTT is a regulator of cargo recognition. The inefficient protein loading or formation of empty autophagosomes leads to the degradation of proteins and even whole organelles [100,101]. Together these data indicate that Huntington’s disease is a classic example of proteinopathies [119]. HTT aggregates in neurons cause cell death, and the toxicity of the aggregates may depend on their intracellular location and stage of the disease [120]. 

However, the role of aggregate formation in the pathophysiology of HD remains uncertain. There is directly opposite evidence confirming that mHTT aggregates are not responsible for pathology, but also provide the cell with a protective mechanism that protects it from the toxic effects of polyglutamine [121,122,123].

During the degradation of improperly folded proteins in the cell, the mutant form of HTT also undergoes such degradation, in particular, it is cleaved by proteases. Thus, an N-terminal fragment containing a long polyglutamine tail is released. These fragments are then transferred to the nucleus, where they affect transcription processes and lead to cell death [88,124,125].

During the development of HD, disruption of gene transcription occurs, as shown on the human post-mortal brain, and samples obtained from transgenic mutant *HTT* mice [125]. HTT binds to many transcription factors, including NF-kB (nuclear factor κB) [126] and p53 [127]. 

Through these interactions, HTT can potentially influence processes such as cell cycle control, apoptosis, stress response, DNA repair, etc. Researchers have suggested that in the nucleus, HTT also functions as a scaffold for various transcriptional complexes [128]. The cytoplasmic pool of HTT can affect transcription by mediating the transport of transcription factors into the nucleus [129]. Besides, HTT itself can also act as a transcriptional cofactor [130] or participate in chromatin rearrangement [131].

Strikingly, HTT not only impairs transport of BDNF but also inhibits its expression in the cortex. These effects are due to the formation of polyglutamine fragments after proteolysis and may be characteristic of other polyglutamine diseases [132]. Additionally, the proteolysis of mHTT leads to the release of toxic C-terminal fragments. The native function of C-terminal fragments is the regulation of dynein-1 activity [133]. The toxic effects of mHTT include the inhibition of chaperones, proteasomal, and autophagic degradation. The participation of mHTT in the physiology of mitochondria, transcription of BDNF, and mitochondrial proteins plays an important role [88]. 

Energy impairment play important role in the pathogenesis of HD. mHTT causes mitochondrial dysfunction. It manifests in decreased mitochondrial biogenesis and trafficking, oxidative stress, impaired mitochondrial calcium handling, ATP deficit, increased apoptosis, and as a result, an energy deficit. Neurons are energy-demanding and susceptible to oxidative stress and energetic failure. 

Changes to several metabolic pathways, for example, glycolysis and tricarboxylic acid cycle have been observed in cell and animal models of HD. Energy and metabolic defects in HD are comprehensively reviewed by [134]. 

Not only the mutant protein but also the mutant RNA of *HTT* can have possible toxic effects [135]. 

In Huntington’s disease, non-functional toxic proteins other than mHTT are generated in neural cells. Their translation is caused by an abnormal secondary structure of mutant CAG-RNA (mCAG-RNA). Atypical translation associated with repeats (repeat associated non-ATG (RAN) translation) leads to the appearance of non-functional peptides and proteins with toxic properties [136]. RAN translation occurs because a hairpin formed in the mRNA by the CAG repeat can initiate the start of translation in the absence of the canonical ATG triplet. In this case, the RAN translation can start from either a non-canonical initiation site or a site within the repeat. In HD, four homopolymer proteins (polyalanine, polyserine, polyleucine, and polycysteine) accumulate in the brain [137]. RAN-translation in HD occurs with sense and antisense chains. In post-mortal sections of the human brain, the largest amounts of these proteins have been found in the caudate nucleus, white matter, and in the case of the juvenile form in the cerebellum. HD-RAN protein accumulation and aggregation is CAG length dependent. Homotypic proteins resulting from RAN translation were also found in a mouse model of HD [137].

Even though *HTT* is expressed in all human tissues, HD is clinically manifested precisely as the degradation of nervous tissue. One of the plausible reasons for this tissue specificity can be just its very own, well-known specific feature of this tissue—the absence of the process of regeneration of nervous tissue in adults. Neurons, as long-living cells, gradually accumulate a mutant protein during their life and subsequently die because stem cells do not restore their population [138]. 

At the same time, such tissue selectivity might arise from a violation of transport processes by affecting specific MT organization in this type of cell. The first event in the development of HD is the death of striatum cells. Researchers assign this to the breach of BDNF transport to the striatum. BDNF is produced in the cortex and then transported along the neuronal axons to the striatum. BDNF is a factor, required for the survival of neurons, and when the transport has affected, cells begin to actively degrade [139].

Thus, several mechanisms lead to mHTT toxicity. At the present stage of research, it is impossible to determine which of these mechanisms makes the most significant contribution to the pathology development. It is likely that for the normal physiology of cells of different tissues types, various functional activities characteristic of normal HTT will be fundamentally important - which means that non-identical toxicity mechanisms can be critical for them. An obvious challenge for further research is the separation of specific toxic properties of mHTT as a substance and effects resulting from the disruption of transport processes. Transport disruption may be due to: (1) defects in the structures that make up the cytoskeleton; (2) broken interconnection and disorganization of the cytoskeletal structures; or (3) defects in motor proteins transporting substances along the cytoskeletal “rails” caused by the physical accumulation of protein aggregates in the cytoplasm of cells.

## 7. The Involvement of HTT in Vesicular Transport and Endocytosis

**Figure 3:**
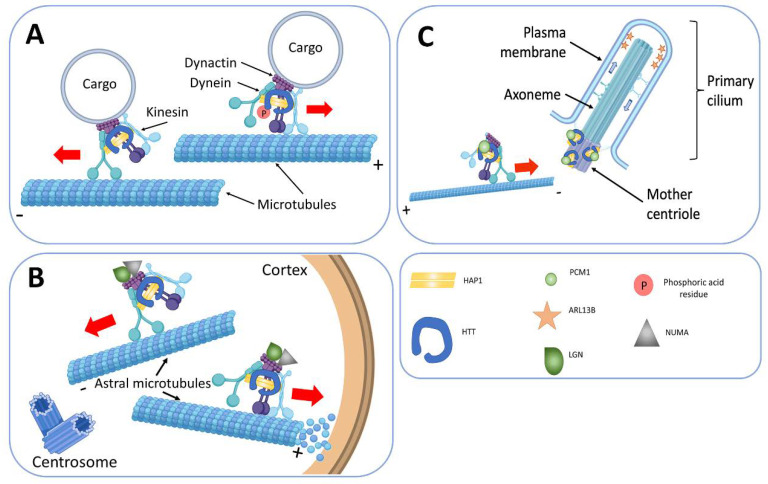
Participation of HTT in microtubule transport. HTT functions as a scaffold for the dynein-dynactin complex. (**A**) Vesicular transport. The phosphorylation of HTT determines the movement direction of the vesicles to the plus or minus end of the MT. (**B**) The formation of the primary cilia. HTT as a part of the dynein dynactin/HAP1 complex transports PCM1 protein to the basal body, which is necessary for the formation and lengthening of the primary cilia. Anterograde and retrograde transport using molecular motors inside the cilia allows the regulation of the number of receptors on its surface, for example, ARL13B, a small G protein from the Ras superfamily specific for the primary cilia. (**C**) Mitotic spindle orientation is adjusted using HTT. During cell division HTT is directed to the spindle poles that determines the accumulation of NUMA and LGN there. In addition, HTT controls NUMA and LGN transport along astral microtubules to the cell cortex. At the cell cortex this complex provides pulling forces on astral microtubules for mitotic spindle orientation. HAP1—huntingtin-associated protein 1; PCM1—pericentriolar material 1 protein; ARL13B—ADP-ribosylation factor-like protein 13B; LGN (GPSM2)—leucine-glycine-asparagine (G-protein-signaling modulator 2); NUMA—nuclear mitotic apparatus protein.

We think that the transport function of HTT is the most important of all investigated so far. HTT controls anterograde and retrograde transport of organelles, including presynaptic vesicles in axons and dendrites of neurons [140]. In this manner autophagosomes [141], endosomes, lysosomes [142,143]; vesicles with BDNF [139]; with APP (amyloid precursor protein) [144,145], and with GABA receptors (γ-aminobutyric acid, GABA) move in the cell [146]. According to several studies, HTT interaction with huntingtin-associated protein 1 (HAP1) regulates the transport. HAP1 binds to the p150Glued subunit of dynactin and the transport protein of the KIF5C kinesin family (Figure 3) [95,96]. HTT also interacts with both N- and C-terminal domains of dynamin-1, serving as a scaffold for this protein [82,133]. HTT increases the speed of vesicle movement along the MTs by binding and retaining glyceraldehyde 3-phosphate dehydrogenase (GAPDH) on the vesicles, which produces energy for axonal transport. Therefore, HTT acts as a scaffold, spatially combining energy production with its consumption [147].

HTT interacts with several proteins involved in endocytosis, including huntingtin-interacting protein 1 (HIP1 and HIP1R). These proteins are involved in membrane invagination processes and the assembly of clathrin-coated vesicles [148,149,150]. Moreover, it also activates the Rab11 GTPase, which, in turn, participates in the transport of vesicles after endocytosis [151]. At the same time, HTT seems to be capable of regulating the switching of vesicle transport from MTs to actin filaments, which is fundamentally important for the development and function of neurons. Such a transition of a vesicle from one component of the cytoskeleton to another can occur as follows: depending on post-translational modifications, HTT can bind to various motor complexes, for example, through the HAP40 protein [42]. After binding of HAP40 to HTT, the complex interacts predominantly with actin, thus switching transport from MTs to actin filaments. Similarly, switching can occur involving the Rab8/optineurin/myosin VI complex [152,153] and the HAP1/dynactin complex [70,95,139]. Switching cargo transport from MTs to actin can occur both near the plasma membrane and near the trans-Golgi—this ensures traffic between the two cell compartments [43].

## 8. HTT and Regulation of the Primary Cilia Formation

The primary cilium is a single immobile organelle found in almost all somatic vertebrate cells [154]. It is a structure surrounded by a specialized cell membrane, consisting of a basal body, an active centriole derivative, and an axoneme growing from it, built of nine pairs of MT doublets. The cilia axoneme can be of considerable length and extend beyond the cell at a different angle to the plane of the substrate [155]. It is plausible that in the body, depending on the type of cells, the primary cilium performs mechano-sensory or receptor functions [156]. Bidirectional transport of pericentriolar material 1 (PCM1) protein, to and from the basal body is necessary for the primary cilium formation. It is tenable that the function of PCM1 is to inhibit the nucleation of MTs [157]. There is molecular evidence of disruption of the primary cilia formation under the influence of mutant HTT. The reason for this effect is the breach of the PCM1 protein transport to the basal body at the base of the cilia. Indeed, in several transformed immortalized human cell cultures, HTT forms a complex with PCM1 and HAP1 and locates in the cell’s centrosome [158].

Several researchers found HTT in the base of cilia in photoreceptor cells, as well as in the axoneme of the primary cilia [158,159,160]. It is credible that the dynein/dynactin/HAP1/HTT complex participates in the transport of the PCM1 protein to the pericentric material (Figure 3). Studies in mice have shown that lack of HTT in cells leads to retrograde transport of PCM1, and the same process has occurred in experiments in Xenopus L. epithelial cells [158,159], and disrupts primary cilia formation. An analysis of the correlation of number and length of primary cilia on cells with the decrease of HTT expression shows that the number of primary cilia decreases and its axoneme abnormally lengthens [158].

## 9. HTT in the Process of Cell Division

In the interphase, HTT locates near the centrosome, and this association depends on the presence of MTs [158]. The expression of HTT is high in actively proliferating cells and throughout mitosis, and it locates at the poles of the spindle of division on astral MTs. During mitosis, HTT moves to the poles of the spindle, together with dynein and provides for the accumulation of NUMA (nuclear mitotic apparatus protein) and leucine-glycine-asparagine (LGN, G-protein-signaling modulator 2, GPSM2) proteins [102,103]. This cortical localization of dynein/dynactin/NUMA/LGN, required to generate pulling forces on astral MTs for mitotic spindle positioning. Hence, loss of HTT function during mitosis leads to spindle misorientation [43]. Studies on cultured fibroblasts from *mHTT* transgenic mice and patients with HD show that mitotic index decreases in *mHTT* cells. A variety of mitotic disturbances has found in experiments: variability in the number of centrioles, a high incidence of aneuploidy and residual body stability [103,161]. 

Therefore, HTT is critically involved in the process of cell division as it participates in many mitotic events. We speculate that HTT can function in cell differentiation processes. In neurogenesis, it can serve as a regulator of maintaining proliferative potential in precursors [102]. Studies show that HTT regulates caspases, proteases, which play an important role in apoptosis, necrosis, and inflammatory processes. HTT can block activation of caspases 3 and 9 [162,163], suggesting that mHTT may contribute to additional cell death by apoptosis.

## 10. Conclusions and Future Outlook

To date, in the result of many studies of the molecular, genetic, and cellular basis of Huntington’s disease, answers to the fundamental questions that have been enigmatic for more than 100 years have received: (1) the gene encoding for the HTT protein has cloned; (2) the mutation, which leads to the expression in the cells a defective protein responsible for this disease has explored; (3) it has found, that HTT participates in many intracellular processes mediated by MTs and other cytoskeletal cell structures.

Actin filaments and MTs and associated proteins play an important role in establishing the morphology of neurons and their plasticity. The dynamic properties of these structures allow them to adapt to changes in the environment and create a structural basis for rapid local intracellular rearrangements and fast neuron shape changes. The ability to stabilize ensures the necessary steadfastness of the formed structures [1]. The results of recent studies demonstrate a credible direct connection of HTT with individual components of the cell cytoskeleton—actin structures and MTs. HTT, as a part of the protein complex dynein/dynactin/HAP1/HTT, participates in directional transport [158]. We hope for a breakthrough in studying the effect of mutant forms of HTT on the processes of transport and cell division in the nearest future.

The role of the cytoskeleton in the development of neurodegenerative processes in humans is not in dispute. Axonal transport disorders are key pathological events contributing to neurodegeneration: polyglutamine diseases, hereditary spastic paraplegia, Charcot-Marie-Tooth disease, amyotrophic lateral sclerosis, and Parkinson’s disease [164]. The researchers found and characterized many genetic mutations in the MT system leading to impaired development of neurons [41]. Studies revealed a whole range of modulators responsible for the development of nervous system pathologies and human diseases. Thus, the universal regulators of MTs and the cytoskeleton as a whole, MT plus-end tracking proteins play an important role in all aspects of the development of the nervous system and involved in the development of many neuropathologies, neurodegenerative and neurological diseases [165]. Therefore, MTs and the actin cytoskeleton appear to be an obvious target in neurodegenerative lesions caused by a mutation leading to HD. 

Studying emerging defects in the cytoskeleton, its components, factors involved in the regulation, and interaction, responsible for intracellular disorders, seems a very promising approach for obtaining answers to pressing questions that we do not have answers to at the moment. This approach will be facilitated by the emergence of new Huntington’s disease models that solve the ethical problems of research. Cell models based on induced pluripotent stem cells differentiated into specialized neural cells that can organize themselves into three-dimensional organoids similar to the complex architecture of the brain [166,167] and model animals have been widely used [168]. These models will allow without breaching ethical standards to study diseases at the organ and organism levels, creating the much-needed theoretical foundations for gene therapy or personalized stem cell therapy that underwent genetic correction [167,169].

## References

[1] Penazzi L., Bakota L., Brandt R. (2016). Microtubule dynamics in neuronal development, plasticity, and neurodegeneration. Int. Rev. Cell Mol. Biol..

[2] Meiring J.C., Akhmanova A. (2020). Microtubules keep large cells in shape. J. Cell Biol..

[3] Alieva I.B. (2014). Role of microtubule cytoskeleton in regulation of endothelial barrier function. Biochemistry.

[4] Dugina V., Alieva I., Khromova N., Kireev I.I., Gunning P.W., Kopnin P.B. (2016). Interaction of microtubules with the actin cytoskeleton via cross-talk of EB1-containing +TIPs and γ-actin in epithelial cells. Oncotarget.

[5] Kopf A., Renkawitz J., Hauschild R., Girkontaite I., Tedford K., Merrin J., Thorn-Seshold O., Trauner D., Häcker H., Fischer K.-D. (2020). Microtubules control cellular shape and coherence in amoeboid migrating cells. J. Cell Biol..

[6] Weisenberg R.C., Broisy G.G., Taylor E.W. (1968). Colchicine-binding protein of mammalian brain and its relation to microtubules. Biochemistry.

[7] Amos L., Klug A. (1974). Arrangement of subunits in flagellar microtubules. J. Cell Sci..

[8] Allen C., Borisy G.G. (1974). Structural polarity and directional growth of microtubules of Chlamydomonas flagella. J. Mol. Biol..

[9] Mitchison T., Kirschner M. (1984). Dynamic instability of microtubule growth. Nature.

[10] Walker R.A., O’Brien E.T., Pryer N.K., Soboeiro M.F., A Voter W., Erickson H.P., Salmon E.D. (1988). Dynamic instability of individual microtubules analyzed by video light microscopy: Rate constants and transition frequencies. J. Cell Biol..

[11] Desai A., Mitchison T.J. (1997). Microtubule polymerization dynamics. Annu. Rev. Cell Dev. Biol..

[12] Shelden E., Wadsworth P. (1993). Observation and quantification of individual microtubule behavior in vivo: Microtubule dynamics are cell-type specific. J. Cell Biol..

[13] O’Brien E.T., Salmon E.D., Walker R.A., Erickson H.P. (1990). Effects of magnesium on the dynamic instability of individual microtubules. Biochemistry.

[14] Drechsel D.N., Hyman A.A., Cobb M.H., Kirschner M.W. (1992). Modulation of the dynamic instability of tubulin assembly by the microtubule-associated protein tau. Mol. Biol. Cell.

[15] Gildersleeve R.F., Cross A.R., E Cullen K., Fagen A.P., Williams R.C. (1992). Microtubules grow and shorten at intrinsically variable rates. J. Biol. Chem..

[16] Hoogenraad C.C., Bradke F. (2009). Control of neuronal polarity and plasticity – a renaissance for microtubules?. Trends Cell Biol..

[17] Conde C., Cáceres A. (2009). Microtubule assembly, organization and dynamics in axons and dendrites. Nat. Rev. Neurosci..

[18] Kapitein L.C., Hoogenraad C.C. (2015). Building the neuronal microtubule cytoskeleton. Neuron.

[19] Kuijpers M., Hoogenraad C.C. (2011). Centrosomes, microtubules and neuronal development. Mol. Cell. Neurosci..

[20] Gotz M., Huttner W.B. (2005). The cell biology of neurogenesis. Nat. Rev. Mol. Cell Biol..

[21] Dehmelt L., Nalbant P., Steffen W., Halpain S. (2007). A microtubule-based, dynein-dependent force induces local cell protrusions: Implications for neurite initiation. Brain Cell Biol..

[22] Witte H., Neukirchen D., Bradke F. (2008). Microtubule stabilization specifies initial neuronal polarization. J. Cell Biol..

[23] Geraldo S., Gordon-Weeks P. (2009). Cytoskeletal dynamics in growth-cone steering. J. Cell Sci..

[24] Yau K.W., Schätzle P., Tortosa E., Pagés S., Holtmaat A., Kapitein L.C., Hoogenraad C.C. (2016). Dendrites in vitro and in vivo contain microtubules of opposite polarity and axon formation correlates with uniform plus-end-out microtubule orientation. J. Neurosci..

[25] Hu X., Viesselmann C., Nam S., Merriam E., Dent E.W. (2008). Activity-dependent dynamic microtubule invasion of dendritic spines. J. Neurosci..

[26] Merriam E.B., Millette M., Lumbard D.C., Saengsawang W., Fothergill T., Hu X., Ferhat L., Dent E.W. (2013). Synaptic regulation of microtubule dynamics in dendritic spines by calcium, F-actin, and drebrin. J. Neurosci..

[27] Baas P.W., Black M.M., A Banker G. (1989). Changes in microtubule polarity orientation during the development of hippocampal neurons in culture. J. Cell Biol..

[28] Baas P.W., Deitch J.S., Black M.M., Banker G.A. (1988). Polarity orientation of microtubules in hippocampal neurons: Uniformity in the axon and nonuniformity in the dendrite. Proc. Natl. Acad. Sci. USA.

[29] Baas P.W., Slaughter T., Brown A., Black M.M. (1991). Microtubule dynamics in axons and dendrites. J. Neurosci. Res..

[30] Brown A., Li Y., Slaughter T., Black M.M. (1993). Composite microtubules of the axon: Quantitative analysis of tyrosinated and acetylated tubulin along individual axonal microtubules. J. Cell Sci..

[31] Stepanova T., Slemmer J., Hoogenraad C.C., Lansbergen G., Dortland B., De Zeeuw C.I., Grosveld F., Van Cappellen G., Akhmanova A., Galjart N. (2003). Visualization of microtubule growth in cultured neurons via the use of EB3-GFP (end-binding protein 3-green fluorescent protein). J. Neurosci..

[32] Kleele T., Marinković P., Williams P.R., Stern S., Weigand E.E., Engerer P., Naumann R., Hartmann J., Karl R.M., Bradke F. (2014). An assay to image neuronal microtubule dynamics in mice. Nat. Commun..

[33] Dent E.W., Gertler F.B. (2003). Cytoskeletal dynamics and transport in growth cone motility and axon guidance. Neuron.

[34] Dent E.W., Kalil K. (2001). Axon branching requires interactions between dynamic microtubules and actin filaments. J. Neurosci..

[35] Van de Willige D., Hummel J.J., Alkemade C., Kahn O.I., Au F.K., Qi R.Z., Dogterom M., Koenderink G.H., Hoogenraad C.C., Akhmanova A. (2019). Cytolinker Gas2L1 regulates axon morphology through microtubule-modulated actin stabilization. EMBO Rep..

[36] Fréal A., Rai D., Tas R.P., Pan X., Katrukha E.A., Van De Willige D., Stucchi R., Aher A., Yang C., Altelaar A.M. (2019). Feedback-driven assembly of the axon initial segment. Neuron.

[37] Dent E.W. (2017). Of microtubules and memory: Implications for microtubule dynamics in dendrites and spines. Mol. Biol. Cell.

[38] Srivastava A.K., Schwartz C.E. (2014). Intellectual disability and autism spectrum disorders: Causal genes and molecular mechanisms. Neurosci. Biobehav. Rev..

[39] Chakraborti S., Natarajan K., Curiel J., Janke C., Liu J. (2016). The emerging role of the tubulin code: From the tubulin molecule to neuronal function and disease. Cytoskeleton.

[40] Stouffer M.A., Golden J.A., Francis F. (2015). Neuronal migration disorders: Focus on the cytoskeleton and epilepsy. Neurobiol. Dis..

[41] Lasser M., Tiber J., Lowery L.A. (2018). The role of the microtubule cytoskeleton in neurodevelopmental disorders. Front. Cell. Neurosci..

[42] Pal A., Severin F., Lommer B., Shevchenko A., Zerial M. (2006). Huntingtin–HAP40 complex is a novel Rab5 effector that regulates early endosome motility and is up-regulated in Huntington’s disease. J. Cell Biol..

[43] Saudou F., Humbert S. (2016). The biology of Huntingtin. Neuron.

[44] Akhmanova A., Steinmetz M. (2008). Tracking the ends: A dynamic protein network controls the fate of microtubule tips. Nat. Rev. Mol. Cell Biol..

[45] Jaworski J., Hoogenraad C.C., Akhmanova A. (2008). Microtubule plus-end tracking proteins in differentiated mammalian cells. Int. J. Biochem. Cell Biol..

[46] Alves-Silva J., Sánchez-Soriano N., Beaven R., Klein M., Parkin J., Millard T.H., Bellen H.J., Venken K.J.T., Ballestrem C., Kammerer R.A. (2012). Spectraplakins promote microtubule-mediated axonal growth by functioning as structural microtubule-associated proteins and EB1-dependent +TIPs (tip interacting proteins). J. Neurosci..

[47] Shi S.-H., Cheng T., Jan L.Y., Jan Y.N. (2004). APC and GSK-3β are involved in mPar3 targeting to the nascent axon and establishment of neuronal polarity. Curr. Biol..

[48] Koester M.P., Müller O., Pollerberg G.E. (2007). Adenomatous polyposis coli is differentially distributed in growth cones and modulates their steering. J. Neurosci..

[49] Eom T.-Y., Stanco A., Guo J., Wilkins G., DesLauriers D., Yan J., Monckton C., Blair J., Oon E., Perez A. (2014). Differential regulation of microtubule severing by APC underlies distinct patterns of projection neuron and interneuron migration. Dev. Cell.

[50] Cao Y., Lipka J., Stucchi R., Burute M., Pan X., Portegies S., Tas R., Willems J., Will L., MacGillavry H. (2020). Microtubule minus-end binding protein CAMSAP2 and kinesin-14 motor KIFC3 control dendritic microtubule organization. Curr. Biol..

[51] Feng C., Thyagarajan P., Shorey M., Seebold D.Y., Weiner A.T., Albertson R.M., Rao K.S., Sagasti A., Goetschius D.J., Rolls M.M. (2019). Patronin-mediated minus end growth is required for dendritic microtubule polarity. J. Cell Biol..

[52] Tucker R.P. (1990). The roles of microtubule-associated proteins in brain morphogenesis: A review. Brain Res. Rev..

[53] Sánchez C., Díaz-Nido J., Avila J. (2000). Phosphorylation of microtubule-associated protein 2 (MAP2) and its relevance for the regulation of the neuronal cytoskeleton function. Prog. Neurobiol..

[54] Dehmelt L., Halpain S. (2004). The MAP2/Tau family of microtubule-associated proteins. Genome Biol..

[55] Chen Q., Zhou Z., Zhang L., Wang Y., Zhang Y.-W., Zhong M., Xu S.-C., Chen C.-H., Li L., Yu Z.-P. (2012). Tau protein is involved in morphological plasticity in hippocampal neurons in response to BDNF. Neurochem. Int..

[56] Fernández-Nogales M., Lucas J.J. (2020). Altered levels and isoforms of Tau and nuclear membrane invaginations in Huntington’s disease. Front. Cell. Neurosci..

[57] Vuono R., Winder-Rhodes S., de Silva R., Cisbani G., Drouin-Ouellet J., Spillantini M.G., Cicchetti F., Barker R.A. (2015). The role of tau in the pathological process and clinical expression of Huntington’s disease. Brain.

[58] Fernández-Nogales M., Cabrera J.R., Santos-Galindo M., Hoozemans J.J.M., Ferrer I., Rozemuller A.J.M., Hernández F., Avila J., Lucas J.J. (2014). Huntington’s disease is a four-repeat tauopathy with tau nuclear rods. Nat. Med..

[59] Blum D., Herrera F., Francelle L., Mendes T., Basquin M., Obriot H., Demeyer D., Sergeant N., Gerhardt E., Brouillet E. (2014). Mutant huntingtin alters Tau phosphorylation and subcellular distribution. Hum. Mol. Genet..

[60] Morales M. (1991). Distribution of acetylated α-tubulin in brain. Cell Tissue Res..

[61] Cambray-Deakin M.A., Burgoyne R.D. (1987). Posttranslational modifications of alpha-tubulin: Acetylated and detyrosinated forms in axons of rat cerebellum. J. Cell Biol..

[62] Robson S.J., Burgoyne R.D. (1989). Differential localisation of tyrosinated, detyrosinated, and acetylated α-tubulins in neurites and growth cones of dorsal root ganglion neurons. Cell Motil. Cytoskelet..

[63] Konishi Y., Setou M. (2009). Tubulin tyrosination is required for the maintenance of neuronal polarity. Neurosci. Res..

[64] Magiera M.M., Bodakuntla S., Žiak J., Lacomme S., Sousa P.M., Leboucher S., Hausrat T.J., Bosc C., Andrieux A., Kneussel M. (2018). Excessive tubulin polyglutamylation causes neurodegeneration and perturbs neuronal transport. EMBO J..

[65] Magiera M.M., Singh P., Gadadhar S., Janke C. (2018). Tubulin posttranslational modifications and emerging links to human disease. Cell.

[66] Akhmanova A., Hoogenraad C.C. (2018). More is not always better: Hyperglutamylation leads to neurodegeneration. EMBO J..

[67] Hayden M.R. (1981). Huntington’s Chorea.

[68] Myers R.H. (2004). Huntington’s disease genetics. J. Am. Soc. Exp. Neurother..

[69] Gusella J.F., Wexler N.S., Conneally P.M., Naylor S.L., Anderson M.A., Tanzi R.E., Watkins P.C., Ottina K., Wallace M.R., Sakaguchi A.Y. (1983). A polymorphic DNA marker genetically linked to Huntington’s disease. Nature.

[70] Li Z., Karlovich C.A., Fish M.P., Scott M.P., Myers R.M. (1999). A putative drosophila homolog of the Huntington’s disease gene. Hum. Mol. Genet..

[71] Macmillan J., Snell R.G., Tyler A., Houlihan G., Fenton I., Cheadle J., Lazarou L., Shaw J., Harper P. (1993). Molecular analysis and clinical correlations of the Huntington’s disease mutation. Lancet.

[72] Bates G.P., Dorsey R., Gusella J.F., Hayden M.R., Kay C., Leavitt B.R., Nance M., Ross C.A., Scahill R.I., Wetzel R. (2015). Huntington disease. Nat. Rev. Dis. Prim..

[73] Tautz D., Schlötterer C. (1994). Simple sequences. Curr. Opin. Genet. Dev..

[74] Baig S.S., Strong M., Quarrell O. (2016). The global prevalence of Huntington’s disease: A systematic review and discussion. Neurodegener. Dis. Manag..

[75] Illarioshkin S.N., Klyushnikov S.A., Seliverstov Y.A. (2018). Huntington’s Disease.

[76] Andrew S.E., Goldberg Y.P., Kremer B., Telenius H., Theilmann J., Adam S., Starr E., Squitieri F., Lin B., Kalchman M.A. (1993). The relationship between trinucleotide (CAG) repeat length and clinical features of Huntington’s disease. Nat. Genet..

[77] Duyao M., Ambrose C., Myers R., Novelletto A., Persichetti F., Frontali M., Folstein S., Ross C., Franz M., Abbott M. (1993). Trinucleotide repeat length instability and age of onset in Huntington’s disease. Nat. Genet..

[78] Holmans P., Massey T.H., Jones L. (2017). Genetic modifiers of Mendelian disease: Huntington’s disease and the trinucleotide repeat disorders. Hum. Mol. Genet..

[79] Sousa C.M., Humbert S. (2013). Huntingtin: Here, there, everywhere!. J. Huntingt. Dis..

[80] Nasir J., Floresco S.B., O’Kusky J.R., Diewert V.M., Richman J.M., Zeisler J., Borowski A., Marth J.D., Phillips A.G., Hayden M.R. (1995). Targeted disruption of the Huntington’s disease gene results in embryonic lethality and behavioral and morphological changes in heterozygotes. Cell.

[81] Zeitlin S., Liu J.-P., Chapman D.L., Papaioannou V., Efstratiadis A. (1995). Increased apoptosis and early embryonic lethality in mice nullizygous for the Huntington’s disease gene homologue. Nat. Genet..

[82] Kaltenbach L.S., Romero E., Becklin R.R., Chettier R., Bell R., Phansalkar A., Strand A., Torcassi C., Savage J., Hurlburt A. (2007). Huntingtin interacting proteins are genetic modifiers of neurodegeneration. PLoS Genet..

[83] Culver B.P., Savas J.N., Park S.K., Choi J.H., Zheng S., Zeitlin S.O., Yates J.R., Tanese N. (2012). Proteomic analysis of wild-type and mutant huntingtin-associated proteins in mouse brains identifies unique interactions and involvement in protein synthesis. J. Biol. Chem..

[84] Tourette C., Li B., Bell R., O’Hare S., Kaltenbach L.S., Mooney S.D., Hughes R.E. (2014). A large scale Huntingtin protein interaction network implicates rho GTPase signaling pathways in Huntington disease. J. Biol. Chem..

[85] De La Monte S.M., Vonsattel J.-P., Richardson E.P. (1988). Morphometric demonstration of atrophic changes in the cerebral cortex, white matter, and neostriatum in Huntington’s disease. J. Neuropathol. Exp. Neurol..

[86] Vonsattel J.P.G., Keller C., del Pilar A.M. (2008). Neuropathology of Huntington’s disease. Handb. Clin. Neurol..

[87] Kremer B., Weber B.H.F., Hayden M.R. (1992). New insights into the clinical features, pathogenesis and molecular genetics of Huntington disease. Brain Pathol..

[88] Ross C.A., Tabrizi S.J. (2011). Huntington’s disease: From molecular pathogenesis to clinical treatment. Lancet Neurol..

[89] Richardson E.P. (1990). Huntington’s disease: Some recent neuropathological studies. Neuropathol. Appl. Neurobiol..

[90] Halliday G.M., McRitchie D., Macdonald V., Double K.L., Trent R., McCusker E. (1998). Regional specificity of brain atrophy in Huntington’s disease. Exp. Neurol..

[91] Rosas H.D., Salat D.H., Lee S.Y., Zaleta A.K., Pappu V., Fischl B., Greve U., Hevelone N., Hersch S.M. (2008). Cerebral cortex and the clinical expression of Huntington’s disease: Complexity and heterogeneity. Brain.

[92] Douaud G., Gaura V., Ribeiro M.-J., Lethimonnier F., Maroy R., Verny C., Krystkowiak P., Damier P., Bachoud-Lévi A.-C., Hantraye P. (2006). Distribution of grey matter atrophy in Huntington’s disease patients: A combined ROI-based and voxel-based morphometric study. NeuroImage.

[93] Myers R.H., Vonsattel J.P., Paskevich P.A., Kiely M.P.H., Stevens B.A., Cupples L.A., Richardson E.P., Bird E.D. (1991). Decreased neuronal and increased oligodendroglial densities in Huntington’s disease caudate nucleus. J. Neuropathol. Exp. Neurol..

[94] Van Der Burg J.M., Bjorkqvist M., Brundin P. (2009). Beyond the brain: Widespread pathology in Huntington’s disease. Lancet Neurol..

[95] Engelender S., Sharp A.H., Colomer V., Tokito M.K., Lanahan A., Worley P., Holzbaur E.L., Ross C.A. (1997). Huntingtin-associated protein 1 (HAP1) interacts with the p150Glued subunit of dynactin. Hum. Mol. Genet..

[96] Caviston J.P., Ross J.L., Antony S.M., Tokito M., Holzbaur E.L. (2007). Huntingtin facilitates dynein/dynactin-mediated vesicle transport. Proc. Natl. Acad. Sci. USA.

[97] Reddy P.H., Shirendeb U.P. (2011). Mutant huntingtin, abnormal mitochondrial dynamics, defective axonal transport of mitochondria, and selective synaptic degeneration in Huntington’s disease. Biochim. Biophys. Acta (BBA) Bioenerg..

[98] Proskura A., Vechkapova S.O., Zapara T.A., Ratushniak A.S. (2017). Protein–protein interactions of huntingtin in the hippocampus. Mol. Biol..

[99] Steffan J.S. (2010). Does Huntingtin play a role in selective macroautophagy?. Cell Cycle.

[100] Martínez-Vicente M., Tallóczy Z., Wong E., Tang G., Koga H., Kaushik S., De Vries R., Arias E., Harris S., Sulzer D. (2010). Cargo recognition failure is responsible for inefficient autophagy in Huntington’s disease. Nat. Neurosci..

[101] Martin D.D.O., Ladha S., Ehrnhoefer D.E., Hayden M.R. (2015). Autophagy in huntington disease and huntingtin in autophagy. Trends Neurosci..

[102] Godin J.D., Colombo K., Molina-Calavita M., Keryer G., Zala D., Charrin B.C., Dietrich P., Volvert M.-L., Guillemot F., Dragatsis I. (2010). Huntingtin is required for mitotic spindle orientation and mammalian neurogenesis. Neuron.

[103] Elias S., Thion M.S., Yu H., Sousa C.M., Lasgi C., Morin X., Humbert S. (2014). Huntingtin regulates mammary stem cell division and differentiation. Stem Cell Rep..

[104] Harjes P., Wanker E. (2003). The hunt for huntingtin function: Interaction partners tell many different stories. Trends Biochem. Sci..

[105] DiFiglia M., Sapp E., Chase K., Schwarz C., Meloni A., Young C., Martin E., Vonsattel J.-P., Carraway R., A Reeves S. (1995). Huntingtin is a cytoplasmic protein associated with vesicles in human and rat brain neurons. Neuron.

[106] Fusco F.R., Chen Q., L’Amoreaux W., Figueredo-Cardenas G., Jiao Y., Coffman J., Surmeier D.J., Honig M.G., Carlock L.R., Reiner A. (1999). Cellular localization of huntingtin in striatal and cortical neurons in rats: Lack of correlation with neuronal vulnerability in Huntington’s disease. J. Neurosci..

[107] McClory H., Wang X., Sapp E., Gatune L.W., Iuliano M., Wu C.-Y., Nathwani G., Kegel-Gleason K.B., DiFiglia M., Li X. (2018). The COOH-terminal domain of huntingtin interacts with RhoGEF kalirin and modulates cell survival. Sci. Rep..

[108] Shin J.-Y., Fang Z.-H., Yu Z.-X., Wang C.-E., Li S.-H., Li X.-J. (2005). Expression of mutant huntingtin in glial cells contributes to neuronal excitotoxicity. J. Cell Biol..

[109] Martin-Aparicio E., Ávila J., Lucas J.J. (2002). Nuclear localization of N-terminal mutant huntingtin is cell cycle dependent. Eur. J. Neurosci..

[110] Hoffner G., Kahlem P., Djian P. (2002). Perinuclear localization of huntingtin as a consequence of its binding to microtubules through an interaction with β-tubulin: Relevance to Huntington’s disease. J. Cell Sci..

[111] Miller J.P., Yates B.E., Al-Ramahi I., Berman A.E., Sanhueza M., Kim E., De Haro M., DeGiacomo F., Torcassi C., Holcomb J. (2012). A genome-scale RNA–interference screen identifies RRAS signaling as a pathologic feature of Huntington’s disease. PLoS Genet..

[112] Shirasaki D.I., Greiner E.R., Al-Ramahi I., Gray M., Boontheung P., Geschwind D.H., Botas J., Coppola G., Horvath S., Loo J.A. (2012). Network organization of the huntingtin proteomic interactome in mammalian brain. Neuron.

[113] Tousley A., Iuliano M., Weisman E., Sapp E., Richardson H., Vodicka P., Alexander J., Aronin N., DiFiglia M., Kegel-Gleason K.B. (2019). Huntingtin associates with the actin cytoskeleton and α-actinin isoforms to influence stimulus dependent morphology changes. PLoS ONE.

[114] Giorgini F. (2013). A flexible polyglutamine hinge opens new doors for understanding huntingtin function. Proc. Natl. Acad. Sci. USA.

[115] Ochaba J., Lukacsovich T., Csikos G., Zheng S., Margulis J., Salazar L., Mao K., Lau A.L., Yeung S.Y., Humbert S. (2014). Potential function for the Huntingtin protein as a scaffold for selective autophagy. Proc. Natl. Acad. Sci. USA.

[116] Rui Y.-N., Xu Z., Patel B., Chen Z., Chen N., Tito A., David G., Sun Y., Stimming E.F., Bellen H.J. (2015). Huntingtin functions as a scaffold for selective macroautophagy. Nature.

[117] Kegel K.B., Kim M., Sapp E., McIntyre C., Castaño J.G., Aronin N., DiFiglia M. (2000). Huntingtin expression stimulates endosomal–lysosomal activity, endosome tubulation, and autophagy. J. Neurosci..

[118] Ravikumar B., Vacher C., Berger Z., E Davies J., Luo S., Oroz L.G., Scaravilli F., Easton U.F., Duden R., O’Kane C. (2004). Inhibition of mTOR induces autophagy and reduces toxicity of polyglutamine expansions in fly and mouse models of Huntington disease. Nat. Genet..

[119] Soto C. (2003). Unfolding the role of protein misfolding in neurodegenerative diseases. Nat. Rev. Neurosci..

[120] Davies S.W., Turmaine M., A Cozens B., DiFiglia M., Sharp A.H., A Ross C., Scherzinger E., Wanker E., Mangiarini L., Bates G.P. (1997). Formation of neuronal intranuclear inclusions underlies the neurological dysfunction in mice transgenic for the HD mutation. Cell.

[121] Arrasate M., Mitra S., Schweitzer E.S., Segal M.R., Finkbeiner S. (2004). Inclusion body formation reduces levels of mutant huntingtin and the risk of neuronal death. Nature.

[122] Miller J., Arrasate M., Shaby B.A., Mitra S., Masliah E., Finkbeiner S. (2010). Quantitative relationships between huntingtin levels, polyglutamine length, inclusion body formation, and neuronal death provide novel insight into huntington’s disease molecular pathogenesis. J. Neurosci..

[123] Miller J., Arrasate M., Brooks E., Libeu C.P., Legleiter J., Hatters D.M., Curtis J., Cheung K., Krishnan P., Mitra S. (2011). Identifying polyglutamine protein species in situ that best predict neurodegeneration. Nat. Methods.

[124] Saudou F., Finkbeiner S., Devys D., E Greenberg M. (1998). Huntingtin acts in the nucleus to induce apoptosis but death does not correlate with the formation of intranuclear inclusions. Cell.

[125] Valor L.M. (2014). Transcription, epigenetics and ameliorative strategies in Huntington’s Disease: A genome-wide perspective. Mol. Neurobiol..

[126] Takano H., Gusella J.F. (2002). The predominantly HEAT-like motif structure of huntingtin and its association and coincident nuclear entry with dorsal, an NF-kB/Rel/dorsal family transcription factor. BMC Neurosci..

[127] Steffan J.S., Kazantsev A., Spasic-Boskovic O., Greenwald M., Zhu Y.-Z., Gohler H., Wanker E., Bates G.P., Housman D.E., Thompson L.M. (2000). The Huntington’s disease protein interacts with p53 and CREB-binding protein and represses transcription. Proc. Natl. Acad. Sci. USA.

[128] Dunah A.W., Jeong H., Griffin A., Kim Y.-M., Standaert D.G., Hersch S.M., Mouradian M.M., Young A.B., Tanese N., Krainc D. (2002). Sp1 and TAFII130 transcriptional activity disrupted in early huntington’s disease. Science.

[129] Marcora E., Gowan K., Lee J.E. (2003). Stimulation of NeuroD activity by huntingtin and huntingtin-associated proteins HAP1 and MLK2. Proc. Natl. Acad. Sci. USA.

[130] Benn C.L., Sun T., Sadri-Vakili G., McFarland K.N., DiRocco D.P., Yohrling G.J., Clark T., Bouzou B., Cha J.-H.J. (2008). Huntingtin modulates transcription, occupies gene promoters in vivo, and binds directly to DNA in a polyglutamine-dependent manner. J. Neurosci..

[131] Seong I.S., Woda J.M., Song J.-J., Lloret A., Abeyrathne P.D., Woo C.J., Gregory G., Lee J.-M., Wheeler V.C., Walz T. (2009). Huntingtin facilitates polycomb repressive complex 2. Hum. Mol. Genet..

[132] Zuccato C., Cattaneo E., Lewin G., Carter B. (2014). Huntington’s disease. Neurotrophic Factors. Handbook of Experimental Pharmacology.

[133] El-Daher M.-T., Hangen E., Bruyère J., Poizat G., Al-Ramahi I., Pardo R., Bourg N., Souquere S., Mayet C., Pierron G. (2015). Huntingtin proteolysis releases non-polyQ fragments that cause toxicity through dynamin 1 dysregulation. EMBO J..

[134] Dubinsky J.M. (2017). Towards an understanding of energy impairment in Huntington’s disease brain. J. Huntingt. Dis..

[135] Bogomazova A.N., Eremeev A.V., Pozmogova G.E., Lagarkova M.A. (2019). The role of mutant RNA in the pathogenesis of Huntington’s disease and other polyglutamine diseases. Mol. Biol..

[136] Zu T., Gibbens B., Doty N.S., Gomes-Pereira M., Huguet A., Stone M.D., Margolis J., Peterson M., Markowski T.W., Ingram M.A.C. (2010). Non-ATG–initiated translation directed by microsatellite expansions. Proc. Natl. Acad. Sci. USA.

[137] Bañez-Coronel M., Ayhan F., Tarabochia A.D., Zu T., Perez B.A., Tusi S.K., Pletnikova O., Borchelt D.R., Ross C.A., Margolis R.L. (2015). RAN translation in Huntington disease. Neuron.

[138] Ramaswami M., Taylor J.P., Parker R. (2013). Altered ribostasis: RNA-protein granules in degenerative disorders. Cell.

[139] Gauthier L.R., Charrin B.C., Borrell-Pages M., Dompierre J.P., Rangone H., Cordelières F.P., De Mey J., E MacDonald M., Leßmann V., Humbert S. (2004). Huntingtin controls neurotrophic support and survival of neurons by enhancing BDNF vesicular transport along microtubules. Cell.

[140] Zala D., Hinckelmann M.-V., Saudou F. (2013). Huntingtin’s function in axonal transport is conserved in drosophila melanogaster. PLoS ONE.

[141] Wong Y.C., Holzbaur E.L. (2014). The regulation of autophagosome dynamics by huntingtin and HAP1 is disrupted by expression of mutant huntingtin, leading to defective cargo degradation. J. Neurosci..

[142] Caviston J.P., Zajac A.L., Tokito M., Holzbaur E.L. (2011). Huntingtin coordinates the dynein-mediated dynamic positioning of endosomes and lysosomes. Mol. Biol. Cell.

[143] Liot G., Zala D., Pla P., Mottet G., Piel M., Saudou F. (2013). Mutant huntingtin alters retrograde transport of TrkB receptors in striatal dendrites. J. Neurosci..

[144] Colin E., Zala D., Liot G., Rangone H., Borrell-Pages M., Li X.-J., Saudou F., Humbert S. (2008). Huntingtin phosphorylation acts as a molecular switch for anterograde/retrograde transport in neurons. EMBO J..

[145] Her L.-S., Goldstein L.S. (2008). Enhanced sensitivity of striatal neurons to axonal transport defects induced by mutant Huntingtin. J. Neurosci..

[146] Twelvetrees A.E., Yuen E.Y., Arancibia-Carcamo I.L., Macaskill A.F., Rostaing P., Lumb M.J., Humbert S., Triller A., Saudou F., Yan Z. (2010). Delivery of GABAARs to synapses is mediated by HAP1-KIF5 and disrupted by mutant Huntingtin. Neuron.

[147] Zala D., Hinckelmann M.-V., Yu H., Da Cunha M.M.L., Liot G., Cordelières F.P., Marco S., Saudou F. (2013). Vesicular glycolysis provides on-board energy for fast axonal transport. Cell.

[148] Engqvist-Goldstein A.E., Warren R.A., Kessels M.M., Keen J.H., Heuser J., Drubin D.G. (2001). The actin-binding protein Hip1R associates with clathrin during early stages of endocytosis and promotes clathrin assembly in vitro. J. Cell Biol..

[149] Waelter S., Scherzinger E., Hasenbank R., Nordhoff E., Lurz R., Goehler H., Gauss C., Sathasivam K., Bates G.P., Lehrach H. (2001). The huntingtin interacting protein HIP1 is a clathrin and alpha-adaptin-binding protein involved in receptor-mediated endocytosis. Hum. Mol. Genet..

[150] Legendre-Guillemin V., Metzler M., Charbonneau M., Gan L., Chopra V., Philie J., Hayden M.R., McPherson P.S. (2002). HIP1 and HIP12 display differential binding to F-actin, AP2, and clathrin. Identification of a novel interaction with clathrin light chain. J. Biol. Chem..

[151] Li X., Sapp E., Valencia A., Kegel K.B., Qin Z.-H., Alexander J., Masso N., Reeves P., Ritch J.J., Zeitlin S. (2008). A function of huntingtin in guanine nucleotide exchange on Rab11. NeuroReport.

[152] Faber P.W., Barnes G.T., Srinidhi J., Chen J., Gusella J.F., E MacDonald M. (1998). Huntingtin interacts with a family of WW domain proteins. Hum. Mol. Genet..

[153] Hattula K., Peränen J. (2000). FIP-2, a coiled-coil protein, links Huntingtin to Rab8 and modulates cellular morphogenesis. Curr. Biol..

[154] Wheatley D. (1996). Expression of primary cilia in mammalian cells. Cell Biol. Int..

[155] Alieva I.B., A Vorobjev I. (2004). Vertebrate primary cilia: A sensory part of centrosomal complex in tissue cells, but a “sleeping beauty” in cultured cells?. Cell Biol. Int..

[156] Pazour G.J., Witman G.B. (2003). The vertebrate primary cilium is a sensory organelle. Curr. Opin. Cell Biol..

[157] Andersen S.S.L. (1999). Molecular characteristics of the centrosome. Nat. Eng. Resist. Plant Viruses.

[158] Keryer G., Pineda J.R., Liot G., Kim J., Dietrich P., Benstaali C., Smith K., Cordelières F.P., Spassky N., Ferrante R.J. (2011). Ciliogenesis is regulated by a huntingtin-HAP1-PCM1 pathway and is altered in Huntington disease. J. Clin. Investig..

[159] Haremaki T., Deglincerti A., Brivanlou A.H. (2015). Huntingtin is required for ciliogenesis and neurogenesis during early Xenopus development. Dev. Biol..

[160] Karam A., Tebbe L., Weber C., Messaddeq N., Morlé L., Kessler P., Wolfrum U., Trottier Y. (2015). A novel function of Huntingtin in the cilium and retinal ciliopathy in Huntington’s disease mice. Neurobiol. Dis..

[161] Sathasivam K., Woodman B., Mahal A., Bertaux F., Wanker E., Shima D.T., Bates G.P. (2001). Centrosome disorganization in fibroblast cultures derived from R6/2 Huntington’s disease (HD) transgenic mice and HD patients. Hum. Mol. Genet..

[162] Rigamonti D., Sipione S., Goffredo D., Zuccato C., Fossale E., Cattaneo E. (2001). Huntingtin’s neuroprotective activity occurs via inhibition of procaspase-9 processing. J. Biol. Chem..

[163] Zhang Y., Leavitt B.R., Van Raamsdonk J.M., Dragatsis I., Goldowitz D., E MacDonald M., Hayden M.R., Friedlander R.M. (2006). Huntingtin inhibits caspase-3 activation. EMBO J..

[164] Millecamps S., Julien J.-P. (2013). Axonal transport deficits and neurodegenerative diseases. Nat. Rev. Neurosci..

[165] Van De Willige D., Hoogenraad C.C., Akhmanova A. (2016). Microtubule plus-end tracking proteins in neuronal development. Cell. Mol. Life Sci..

[166] Nekrasov E.D., Vigont V.A., Klyushnikov S., Lebedeva O.S., Vassina E.M., Bogomazova A., Chestkov I.V., Semashko T.A., Kiseleva E., Suldina L.A. (2016). Manifestation of Huntington’s disease pathology in human induced pluripotent stem cell-derived neurons. Mol. Neurodegener..

[167] Csobonyeiova M., Polak S., Danišovič L. (2020). Recent overview of the use of iPSCs Huntington’s disease modeling and therapy. Int. J. Mol. Sci..

[168] Dvorzhak A., Grantyn R. (2020). Single synapse indicators of glutamate release and uptake in acute brain slices from normal and Huntington mice. J. Vis. Exp..

[169] Chen Y., Carter R.L., Cho I.K., Chan A.W.S. (2014). Cell-based therapies for Huntington’s disease. Drug Discov. Today.

